# Do VIP medical services damage efficiency? New evidence of medical institutions’ total factor productivity using Chinese panel data

**DOI:** 10.3389/fpubh.2023.1261804

**Published:** 2024-01-24

**Authors:** Yan Yang, Mingwang Cheng, Ning Chen, Ling Yuan, Zhaoxin Wang

**Affiliations:** ^1^School of Economics and Management, Tongji University, Shanghai, China; ^2^School of Public Health, Shanghai Jiao Tong University School of Medicine, Shanghai, China; ^3^Department of Dermatology, The Fifth People’s Hospital of Hainan Province, Hainan Medical University, Haikou, China; ^4^School of Management, Hainan Medical University, Haikou, China

**Keywords:** VIP medical services, total factor productivity, Global Malmquist index, technological advances, pure technical efficiency

## Abstract

This study examines the causal impact of very important person (VIP) medical services on hospital total factor productivity in Deyang, a prefectural-level city in western China, spanning the years 2015–2020. This aims to offer empirical evidence and policy recommendations for the implementation of VIP practices in the medical field. A secondary unbalanced panel dataset of 416 observations was collected from the annual reports of the Health Commission and 92 eligible medical institutions were included. This study utilized a two-stage strategy. First, the Global Malmquist index was used to calculate the total factor productivity and its decomposition terms for hospitals from 2015 to 2020. In the second stage, two-way fixed effects models and Tobit models were used to identify the relationship between VIP medical services and hospital efficiency; instrumental variables were used to solve potential endogeneity problems in the model. The results showed that VIP medical services had a significantly negative impact on medical institutions’ efficiency. The technological advances and pure technical efficiency related to VIP medical care may help explain these negative impacts, which were heterogeneous across groups divided by the nature of the hospital and the outside environment. It is imperative to prioritize the standardized provision of VIP medical services for medical institutions, optimize management and service process, enhance the training of clinical and scientific research capabilities of medical personnel, and scientifically allocate resources for both VIP and general medical services. This will help mitigate health inequality while improving the overall quality of medical services.

## Introduction

The very important person (VIP) phenomenon within the healthcare industry raises discussions about equity and efficiency in healthcare service delivery. VIP healthcare services often include better access to healthcare resources, special attention from staff, and luxurious amenities (1). Additionally, with the promotion of the VIP healthcare model worldwide, the target of VIP services in some medical institutions is not limited to celebrity patients or potentates (2), but such services are open to all who are willing to pay the extra price (3, 4). In the US, doctors (usually internists or family physicians) provide medical care to patients who pay for annual, semi-annual, quarterly, or monthly appointments (5, 6), which is called concierge medicine (also called retainer practice or consumer-focused care). In China, with permission from the medical regulator, hospitals with some comprehensive strengths opened special needs medical services or VIP medical services, including outpatient or ward services (7). VIP and concierge medical services are forms of medical service differentiation with limited scale (the pricing and scale of VIP services offered by public hospitals are restricted), providing personalized medical services such as comprehensive annual check-ups, preventative care, same-day appointments, and rapid medical response times (1, 8). For healthcare providers, such services offer greater autonomy in the care of more manageable patients, address concerns about heavy workloads for physicians, and improve income in response to declining wages due to increasingly reduced reimbursements (3, 9, 10). For patients, these services provide more immediate, convenient, and individualized access to care, which bridges the gap between patient empowerment and patient engagement (9, 11, 12). However, VIP models have associated ethical and legal concerns, as some public health experts believe them to be elitist and worry that their widespread implementation can lead to the under-or over-utilization of healthcare resources (1, 4, 13). In developing countries, where health resources are often insufficient to meet demands when medical institutions provide both basic and VIP medical services, hospital efficiency is likely to be compromised. This study investigates the effects of VIP medical services on the efficiency of medical institutions in China, one of the largest developing nations.

The ethics and application prospects of VIP medical services are of academic concern, although the literature contains inconsistent conclusions. Most studies report that VIP medical services may have a positive impact on all patients and service providers if implemented properly, especially in the face of significant demand for medical care by health insurance companies and aging populations. Others posit that such services address burnout among doctors (10). For example, concierge medicine has great prospects in primary medical care, medical beauty, and orthodontics, which is a developmental model worth exploring in the future (6). VIP floors in China are financially successful, bringing additional revenue to the general hospital funds deficits caused by public interest positioning (3). However, some researchers have argued that this form of medical delivery harms equity and efficiency in healthcare (3, 12, 14). For example, several scholars believe that concierge medical treatment is only accessible to some people based on wealth and health status, and does not improve health status or level of care (15, 16). Additionally, studies have explored the development status and factors that influence VIP medicine or concierge medicine (3, 17, 18); however, the causal relationship between VIP care and efficiency remains unclear. Furthermore, empirical evidence from developing countries is scant as VIP medical services originated in developed countries. As a developing country with a large population base, the heterogeneous demand for medical environments in China provides a large market and practice sample for VIP medicine. If VIP medicine is treated differently as a risk factor for the efficiency of medical institutions in developing countries, the impact might be different.

China’s rapid economic development has improved living standards and created more diverse medical demand. Additionally, the uneven development of medical service tiers has made discussion of the rationality of VIP medicine increasingly contentious. In Shanghai, the number of public hospitals that provide VIP medical services increased from 32 in 2016 to 43 in 2019. In terms of outpatient revenue, the overall revenue of VIP medical services grew at a compound annual growth rate of 18.5% (19). Due to the COVID-19 pandemic in 2020, nearly 4,000 of China’s 9,000 public hospitals experienced losses exceeding 60 billion yuan from January to September (20). VIP medicine, because of its high price positioning, is considered a special compensation channel for public hospitals in China (3). The debate on its existence and abolition has triggered new discussions. Nevertheless, whether, how, and why VIP medicine affects medical institution efficiency is not clear. As such, there remains a lack of empirical evidence on the future practice of VIP medicine in China.

Since the 1980s, Chinese hospitals have changed significantly in the market economy era. Simultaneously, China has carried out several medical and health reforms to ensure responsiveness to residents’ demand for medical treatment. During this period, reform measures such as expert clinics, family sick beds, Senior Cadres Wards (wards established for leading cadres with higher professional titles or military ranks before retirement), and designated doctor surgeries, in which patients pay a fee to specify the surgeon, have been promoted and implemented in public hospitals. In 1993, the Chinese government officially allowed medical institutions to provide VIP medical services while ensuring the provision of basic services, with fees fluctuating according to demand. Driven by the pursuit of profit, medical institutions actively apply to establish VIP medical services. However, as standards and supervision are lacking, the basic medical resources of some public hospitals with VIP medical services have been crowded out. Hence, the practice of VIP medical services in China has been restricted. In 2013, the Chinese government proclaimed support for private capital to run hospitals. As the market economic system improved, so did health medical resources, and VIP medicine returned to the public eye.

Compared with the practice of concierge medicine in developed countries, VIP medical services in China are still immature. It is necessary to research the survival and abolishment of VIP medical services, especially in China, where medical resources are not balanced and sufficient. In previous studies, some scholars have argued that VIP services pose unique moral and value-based challenges for healthcare providers (15). On the one hand, in the VIP medical customer group, markets have evolved to produce a range of products that offer higher quality and amenities to those with sufficient willingness to pay (21). It is often the rich, not the more severe, who get VIP medical care. However, previous studies have not shown clear evidence of improved health among these people (22). Instead, as some doctors turn to VIP medical care with less reception volume, the general medical market becomes more crowded, which increases the probability of resource misallocation (23). Additionally, some doctors who provide VIP medical care posit that VIP patients may require unnecessary medical examination or care, which will affect doctors’ clinical decisions and lead to low efficiency or even waste of medical resources (23). In summary, medical institutions offering VIP medical services may experience a negative impact on their operational efficiency through certain mechanisms. While similar work in understanding the determinants of hospital efficiency exists in the contexts of developed countries (24–26), few studies have examined the underlying forces for productivity changes in response to changes in healthcare delivery (27). While the control factors that explain efficiency variation or productivity growth are data dependent, the panel nature of our dataset allows us to estimate a second-stage regression with controlled factors such as outside environment characteristics, hospital characteristics, medical institution policies, and so forth.

This study makes two major contributions to the literature. First, the causal effects of VIP medicine on the operational efficiency of healthcare facilities are estimated. One difficulty in identifying the causal relationship between VIP medicine and medical institutions’ operational efficiency is potential reverse causality. For example, medical institutions with higher numbers of professional doctors or departments have more advantages in conducting VIP medical practices given their reputation (28). Private specialty hospitals not covered by basic social medical insurance does not cover, such as those for cosmetic medicine and orthodontic dentistry, are more likely to provide VIP medical services for profit (29). If these factors covaring with VIP medicine practice cannot be measured directly, the estimate would be biased. The instrumental variable (IV) method is adopted to address this endogeneity problem, using the number of key departments (i.e., key clinical specialties) in preparation as an instrumental variable for whether a VIP bed is applied, and the proportion of transportation land area and total hospital income as instrument variables for the ratio of VIP beds. To the best of our knowledge, this study is the first to estimate the causal effects of VIP medical practices on institutions’ operational efficiency in a developing country.

Second, the mechanisms through which VIP medical services affect hospital efficiency are explored. Contrary to the expectation that satisfying patients’ diverse medical service demands and improving doctors’ financial incentives will increase the productivity of medical institutions, the analysis of total factor productivity (TFP) and its decomposition items show that the overall TFP of hospitals worsens when it provides VIP medical services. First, VIP medical treatment leads to mismatches between doctors and patients. It also tends to reduce the efficiency of resource utilization and management and decreases the pure technical efficiency of medical services. Second, financial incentives for VIP care make hospitals dependent on technology and equipment, which impedes the technical progress of medical workers.

The remainder of this paper is organized as follows: Section 2 introduces the data and identification strategy. Section 3 reports the main results and heterogeneity analysis. Section 4 summarizes the conclusions.

## Methods

### Data

The empirical analysis identified the effect of VIP medical practices on medical institutions’ operational efficiency. Accordingly, 582 annual reports of 125 medical institutions for the period of 2015–2020, provided by Deyang (a prefecture-level city in West China Sichuan Province) Health Commission, were analyzed.

The Deyang Medical Institutions Annual Report is compiled by medical institutions at the request of the Health Commission to track the development of the health system during the 13th Five-Year Plan. This dataset contains rich information on the characteristics of all local medical institutions, including staffing setup, asset status, the number of medical services provided, and other operational data from 2015 to 2020. The number of VIP hospital beds listed in the dataset was used as a proxy for the degree of VIP medical practices. Medical institution characteristics, which were used as control variables in the regression, were also obtained from the dataset. Observations with missing or inaccurate data were excluded, as were data from medical institutions with only one or two records in the TFP calculation. Various consistency checks were performed on the data to ensure that changes in the mean and distribution of values over time were not excessive. After eliminating the observations described above, an unbalanced panel dataset of 416 observations (92 medical institutions) was obtained.

Additionally, environmental data were obtained as control variables at the district level using the Deyang City Statistical Yearbook for 2015–2020, as well as related data on policy changes from the government website.

### Key variables

#### Total factor productivity

The main outcome of interest was the operational efficiency of medical institutions, and TFP is a common proxy variable used in previous studies. TFP change refers to changes in outputs that cannot be attributed to shifts in inputs. As medical institutions have multiple inputs and outputs, non-parametric methods represented by data envelopment analysis (DEA) are commonly used to measure the TFP of medical institutions (30–32). DEA often uses the Malmquist productivity index; however, this conventional approach may be biased due to infeasibility and a lack of circularity. The Global Malmquist productivity index proposed by Pastor and Lovell (33) was used to overcome these issues regarding panel-analyzed data, which can measure dynamic changes in the production efficiency of decision-making units in different periods (34, 35). The Global Malmquist productivity index is also useful when the unbalanced panel and data points belonging to different frontiers do not correlate (36). The processed balanced panel data was analyzed in the robustness test to ensure the consistency of the conclusions. The FGNZ decomposition method (37) was used to obtain the changes in TFP and its decomposition terms, including Technical Change (TECHCH), Pure Efficiency Change (PEFFCH), and Scale Change (SCH). The Global Malmquist index in period t is the ratio of two distance functions measuring the maximal proportional change of the input–output combination of the previous period ( $x_{t-1}$ , $y_{t-1}$ ) (denominator) and current period ( $x_{t}$ , $y_{t}$ ) (numerator), holding the production technology of a certain point of time constant (26). $TFP_{t}^{MQ}$ , TFP change (TFPCH) is the geometric mean of two Global lmquist indices for production technology in period *t-1* and *t*, as presented in Equation 1:


(1) $$\begin{array}{l} TFP_{t}^{MQ}=MQ_{t}\left(x_{t},y_{t},x_{t-1},y_{t-1}\right)=\\ {\left[\left(\frac{D_{t-1}\left(x_{t},y_{t}\right)}{D_{t-1}\left(x_{t-1},y_{t-1}\right)}\right)\left(\frac{D_{t}\left(x_{t,}y_{t}\right)}{D_{t}\left(x_{t-1},y_{t-1}\right)}\right)\right]}^{\frac{1}{2}}=\\ \mathrm{TECHCH}\left(x_{t},y_{t},x_{t-1},y_{t-1}\right)*\mathrm{PEFFCH}\\ \left(x_{t},y_{t},x_{t-1},y_{t-1}\right)*SCH\left(x_{t},y_{t},x_{t-1},y_{t-1}\right)\; \end{array}$$

Since $TFP_{t}^{MQ}$ is the rate of change of TFP compared with the previous year—that is, the TFP level is obtained by multiplying measured TFPCH—which cannot be directly used for regression of the econometric model, it must be transformed accordingly. Therefore, referring to the mainstream practice of the existing literature (38), the medical institution’s TFP level in 2015 was assumed to be 1 (this hypothesis will be verified in the subsequent robustness test section); then, the TFP level in 2016 is the TFP level in 2015 multiplied by the $TFP_{t}^{MQ}$ in 2016. By analogy, the medical institution’s TFP from 2016 to 2020 was obtained. TECHCH, PEFFCH, and SCH were treated in the same way to obtain Technical (TECH), Pure Efficiency (PEFF), and Scale (SC), respectively.

#### Input and output

The measurement of hospital TFP varies substantially in the selection of input and output indicators in previous studies (30, 34, 39). To ensure the efficiency of DEA, indicators of inputs and outputs were selected based on mainstream literature. Considering the requirements of the DEA model for the number of decision-making units and data representation, availability, stability, and independence, the following variables were selected as input indicators: number of administrators, number of licensed physicians (including assistants), number of registered nurses, number of other health workers, net assets, and the architectural area of buildings. Among the indicators, the input of the medical and health service system primarily consists of material capital and labor force (40). The four types of labor inputs were the hospital’s labor force; net assets and the architectural area of buildings were considered the hospital’s physical capital. Net assets and architectural area can be characterized by indicators such as the beds and equipment of medical institutions, which can also overcome the efficiency differences caused by different levels of medical service institutions.

The output of health institutions are measured primarily in terms of abstract goals, such as curing disease and restoring the physical health of patients (41), which focus on diagnosis and treatment services and inpatient services (40). Therefore, the number of outpatient and emergency patients, the number of discharged patients, and the number of operations for discharged patients relating to the hospital’s service capacity were selected as the output indicators (40, 42, 43). As China’s hierarchical medical system is imperfect, emergency services are an important component of the output of medical institutions. With a small sample, highly correlated indicators should be combined to ensure the representativeness of the indicators. Therefore, as in Kok and Ng (31), the outpatient service volume and emergency service volume of the hospitals were combined into the number of outpatient and emergency patients. Ideally, the total number of inpatients should be reported in either the case-mix index or the diagnosis-related group system. However, such information was not available in the dataset. Therefore, the number of inpatients was used.

#### Proxy variable for the degree of VIP medical services

To determine the degree of VIP medical services in the sample hospitals, the availability of VIP beds, the ratio of VIP beds, and the number of VIP beds (logarithm) were selected as proxy variables.

### Identification strategy

#### Baseline fixed-effects empirical strategy

The TFP derived from the Global Malmquist index model was used as the dependent variable in the second stage of analysis. Similar to Karmann and Roesel (26), using fixed-effects estimation helped mitigate endogeneity problems arising from time-invariant unobserved characteristics and individual fixed-effects, which cover unobservable heterogeneity across sample hospitals (i.e., the scale of the hospital). Time-fixed effects were used to eliminate the effects of national reforms that affected all districts and counties simultaneously. The logarithms of all control variables were taken to ensure the stationarity and heteroskedasticity of robust standard errors (44, 45). All data analysis in this research was conducted using Stata16.0 software. Our ordinary least squares (OLS) model was as in Equation 2:


(2) $$TFP_{i,t}=a_{i}+d_{t}+P_{i,t}\beta +Z_{i,t}\gamma +e_{i,t}$$

where $TFP_{i,t}$ represents the productivity of hospital i in period t . αi and $d_{t}$ define individual fixed effects and year fixed effects. $e_{i,t}$ is the error term. *P* represents proxy variables of the degree of VIP medical services applied.

A hospital’s operational efficiency is not only affected by factor input and technological progress, but also by many environmental factors such as economic strength, population differences, government support, and the quality of hospital staff (46, 47). To ensure the comprehensive selection of environmental variables, control variables ( $Z_{i,t}$ ) were included that covered three aspects: socio-economic environment, external characteristics of medical institutions, and policy induction of medical institutions (26). The following socio-economic environment variables that may portray regional hospital demand were selected: regional *per capita* GDP (46), population density (40, 48), urban unemployment rate (49), population mortality (to characterize the level of medical technology), and government attention to health (50). Additionally, to prevent endogeneity problems caused by missing variables and reduce the heterogeneity of different hospital sizes, governmental subsidies, ownership, specialization, profit or not, medical students training base or not, support health insurance or not, rank, capital intensity (ratio of net assets and total staff), number of hospital beds, number of key departments, and equipment value above 10,000 yuan (to capture the level of technological progress of medical service institutions) were assessed. Finally, to capture health service utilization or overcrowding, medical institution policy-induced variables (the ratio of nurses to physicians, ratio of beds to nurses, bed occupancy rate (number of beds occupied divided by the average number of beds available), average length of stay for discharged patients, and the average number of patients physicians diagnosed) were considered to capture health service utilization or overcrowding. All control variables other than the dummy variables were in logarithmic form. To exclude the effects of price levels, all financial data were deflated by the 2015 GDP-based price index before regression.

#### Panel data Tobit empirical strategy

The distribution of TFP cannot be less than zero, which is typical of censored data. Additionally, a consistent estimator may not be obtained if the OLS method is used for its estimation (51). This may make models in Section 2.4.1 inappropriate and may necessitate the use of a corner solution model; that is, the censored Tobit regression model, in which the maximum likelihood estimation method is used to estimate the censored data (52). Sufficient data was lacking to estimate the fixed effect using the likelihood method, so semi-parametric estimation was employed. Moreover, the specific form of the pseudo residual is not required, and a consistent estimator can be obtained even in the case of heteroscedasticity of interface individuals (53). The fixed-effects Tobit model for estimation was used. Comparing the two-way FE model, panel Tobit estimation results can also support the robustness of the research conclusions.

#### Instrumental variables strategy

To identify the causal effect of VIP medical services on hospital efficiency, endogeneity needed to be addressed, which arises primarily from the reverse causality between VIP services and hospital TFP. For example, healthcare providers with more efficient operations may be more likely to apply for and successfully set up VIP medical services (7). Additionally, some unobservable aspects, such as certain factors of production, may also cause an endogeneity bias. For instance, the advanced medical technicians that affect the outside evaluation of a medical institution may affect both VIP medical services and hospital efficiency (54). Thus, the OLS estimate may experience upward bias if such unobserved variables improve the chances of applying for VIP medical services. To address the possible endogeneity, a two-stage least squares (2SLS) analysis was used to examine the causal impact of VIP medical services and hospital efficiency.

The number of key departments in preparation was employed as the instrumental variable. Specifically, the number of key departments in preparation at the end of the year obtained in the hospital’s annual report was used. Key departments indicate overall strength in clinical medicine, teaching, scientific research, breakthroughs, and innovations in a specialized field. In general, hospitals will choose departments with relatively high levels of technical and professional quality to prepare and construct key departments (55). Typically, the department undergoes a thorough external evaluation and is likely to develop into a brand department that provides VIP medical services. Thus, the number of key departments in preparation satisfies the correlation conditions; the strength of the correlation will be assessed in later estimations. Exclusion restrictions should also be satisfied for a valid IV. All key departments under construction in our sample received financial subsidies from superior authorities in the current year; however, they cannot be used as additional input factors to affect the operating efficiency of medical institutions in that year. First, according to the existing literature, the input and output of key departments under construction have not achieved optimal comprehensive technical efficiency due to diseconomies of scale (56). It is supposed that this does not affect the overall operating efficiency of medical institutions. Second, the construction of key departments primarily focuses on optimizing process management and improving the scientific research skills of medical staff (55, 57), which is a long-term process. Substantially, it is conservatively believed that the key department in preparation will not affect the TFP of the hospital in a short period.

Similar to the selection criteria of the abovementioned instrumental variables for whether to set up VIP medical care, the proportion of transportation land area and total hospital income were selected as the instrumental variables for the number of VIP beds. Geographical location and current financial status are important factors that affect hospital service innovation (58); however, no evidence indicates their relationship to hospitals’ TFP.

## Results

### Descriptive statistics

In our sample, hospitals with VIP services appeared to have better factors of production and service output. The sample included 40 hospitals with VIP services and 376 hospitals without VIP services: the opening ratio of VIP services (9.62%) conforms to medical authority requirements, as it is below 10%. In Table 1, the outside environment characteristics, external characteristics, policy-induced variables, internal characteristics, and performance characteristics of hospitals with and without VIP services were compared. Hospitals with VIP services are in areas with better economic status than hospitals without VIP services, and are more likely to be public, non-profit, high-grade, and mass hospitals. Although the resource endowments and performance of hospitals with VIP services are better than those without VIP services, indicators such as the ratio of nurses to physicians and the ratio of beds to nurses indicate that hospitals without VIP services are more well-resourced. The significant differences between these two types of hospitals also suggest the possibility of endogeneity, which will be analyzed later.

**Table 1:** Sample statistics for hospitals, 2015–2020.

	Hospitals with VIP service		Hospitals without VIP service		Mean Diff	*T*-value
	Mean	St. dev.	Mean	St. dev.		
Outside environment characteristics						
Regional GDP *per capita* (CNY)	70345.730	11652.820	66056.610	15417.450	4289.120	1.708*
Population density (people/km2)	760.883	336.436	812.135	294.192	−51.252	−1.033
Urban unemployment rate (%)	3.725	0.151	3.782	0.144	−0.057	−2.362**
Population mortality (%)	9.750	3.770	9.621	4.142	0.129	0.189
Government attention to health^a^	14.625	4.106	14.210	3.637	0.415	0.677
Medical institution’s external characteristics						
Governmental subsidy	0.075	0.084	0.060	0.127	0.015	0.725
Ownership (Public hospital = 1, Private hospital = 0)	0.700	0.464	0.479	0.500	0.221	2.677***
Specialization (Specialized hospital = 1, otherwise = 0)	0.275	0.452	0.375	0.485	−0.100	−1.248
Profit or not	0.150	0.362	0.399	0.490	−0.249	−3.120***
Medical students’ training base or not	0.225	0.423	0.024	0.153	0.201	6.196***
Support health insurance or not	1.000	0	0.957	0.202	0.043	1.330
Rank	2.400	0.744	1.420	0.854	0.980	6.977***
Capital intensity (ratio of total assets and total staff)	130.544	83.555	101.722	99.983	28.822	1.758*
Number of hospital beds	427.475	349.386	173.582	202.950	253.893	6.910***
Number of key departments	2.425	4.018	0.638	1.323	1.787	6.094***
Equipment value above 10,000 yuan (10^4^ CNY)	6523.475	5787.493	1416.875	2812.916	5106.600	9.557***
Average outpatient cost (10^4^ CNY)	0.179	0.054	0.199	0.179	−0.020	−0.688
Medical institution policy-induced						
Ratio of nurses to physicians	0.628	0.073	0.884	0.522	−0.256	−3.093***
Ratio of beds to nurses	2.173	1.265	3.569	2.746	−1.396	−3.177***
Beds occupancy rate (%)	38.637	11.287	24.224	19.344	14.413	4.626***
Average length of stay for discharged patients	8.596	1.965	23.311	59.174	−14.715	−1.571
Average number of patients physicians diagnosed	2406.695	639.480	1719.358	1326.486	687.337	3.235***
Medical institution internal characteristics (Input)						
Net assets (10^4^ CNY)	166317.8	224,145	40373.850	64722.320	125943.950	8.201***
Number of administrators	21.575	22.932	7.638	11.509	109.586	8.926***
Number of licensed physicians (including assistants)	156.575	149.048	46.989	60.871	172.223	9.395***
Number of registered nurses	239.625	218.877	67.402	91.813	13.937	6.436***
Number of other health workers	72.100	49.657	25.266	30.338	46.834	8.625***
Building area^b^ (km^2^)	31209.600	25080.94	10555.270	15501.220	20654.330	7.463***
Medical institution performance characteristics (Output)						
Number of outpatient and emergency patients	351949.300	319027.800	90675.560	141592.100	261273.740	9.431***
Number of discharged patients	16488.800	14964.860	4292.032	6858.680	12196.768	9.188***
Number of operations for discharged patients	5865.075	6269.301	1153.468	2149.197	4711.607	10.088***
Outpatient and emergency mortality (%)	0.003	0.008	0.005	0.018	−0.002	−0.806
Hospital mortality (%)	0.605	0.574	0.619	1.038	−0.014	−0.083
Number of medical disputes	23.000	44.441	2.699	11.831	20.301	6.901***
Instruments variables						
Number of key departments in preparation	0.800	1.305	0.114	0.479	0.686	6.793***
Proportion of transportation land area	0.030	0.011	0.028	0.009	0.002	1.450
Total hospital income (10^8^ CNY)	17.270	20.235	4.081	7.336	13.190	8.487***
Observations	40		376			

The table shows the summary statistics of hospitals with VIP service and hospitals without VIP service.

All data are raw and untransformed.

*p < 0.1, **p < 0.05, ***p < 0.01.

^a^The government’s attention to health was measured primarily through a manual reading of a large number of medical and health policy texts, extracting keywords including medicine, medical insurance, hospitals, sanitation, infectious diseases, medical treatment, and drug costs that can better capture information related to medical and health policy fields. On this basis, the contents of the government work reports of districts and counties over the years were retrieved and matched. The frequency of precise sentences containing keywords was used to measure the intensity of local government’s attention allocation in the medical and health field.

^b^Building area refers to the total area including service and administration areas.

Table 2 reports the computed TFP and its three components, TECH, PEFF, and SC. These figures are yearly geometric means of the sampled hospitals, following multiplicative transformation. In the early stages (2015–2018), hospitals that offered VIP services appeared to have better productivity performance than those that did not. However, in 2017–2019, the TFP of the two groups shows the opposite trend: the TFP of hospitals with VIP services decreased year by year, while that of hospitals without VIP services increased year-on-year. The apparent decrease in TFP of both groups in 2019–2020 may be due to the impact of the COVID-19 outbreak. The comparison of 2016 and 2019 data shows that the decrease in the TFP of hospitals offering VIP services is primarily attributed to the decrease of PEFF, which is consistent with the *t*-test results of different groups regarding medical disputes in Table 1. Hospitals that providing VIP medical services face more medical disputes than those that do not. Another difference between the groups is that the production frontier (TECH) is formed by the sample of hospitals with VIP services in the sample period; however, hospitals without VIP services attempted to catch up with the production frontier in some years (2017–2018 and 2019–2020). In other words, hospitals with VIP services seem to be the technology leaders of the sample. This is consistent with Kok and Ng’s (31) conclusion on public and non-public hospitals. Most VIP medical services are established in public hospitals that were in the position of being caught up. Technical deterioration (TECH decline) and technological progression (TECH rise) are found in hospitals with VIP services. Meanwhile, hospitals without VIP services showed steady technological progress in most years. The t-test results of medical institutions providing VIP services (*n* = 40) and those not providing VIP medical services (*n* = 376) shows a significant difference in TFP values between the two groups (*T*-value = 1.856, *p* < 0.05), and the mean TFP values of hospitals providing VIP services (mean = 1.337, std. = 1.081) are higher than those of medical institutions that not providing them (mean = 1.112, std. = 0.668).

**Table 2:** Productivity results, 2015–2020.

	Hospitals with VIP service		Hospitals without VIP service		Mean diff
	Mean	St. dev.	Mean	St. dev.	
2015–2016					
TFP	1.421	0.905	1.042	0.311	0.379
PEFF	1.226	0.377	0.999	0.293	0.227
TECH	1.106	0.257	1.044	0.202	0.062
SC	0.977	0.056	1.020	0.130	−0.043
Observations	4		69		
2016–2017					
TFP	1.483	1.431	1.129	0.641	0.354
PEFF	1.126	0.464	1.020	0.572	0.106
TECH	1.173	0.434	1.096	0.367	0.076
SC	0.969	0.064	1.052	0.256	−0.083
Observations	6		67		
2017–2018					
TFP	1.345	1.247	1.240	0.753	0.105
PEFF	1.052	0.432	1.074	0.632	−0.022
TECH	1.128	0.382	1.132	0.461	−0.004
SC	1.002	0.063	1.059	0.277	−0.057
Observations	8		62		
2018–2019					
TFP	1.308	1.179	1.325	0.907	−0.017
PEFF	1.056	0.412	1.151	0.786	−0.095
TECH	1.127	0.359	1.124	0.376	0.004
SC	0.984	0.106	1.051	0.249	−0.067
Observations	9		56		
2019–2020					
TFP	1.268	1.203	1.294	0.866	−0.026
PEFF	1.058	0.416	1.095	0.663	−0.037
TECH	1.134	0.360	1.143	0.382	−0.009
SC	0.952	0.153	1.036	0.249	−0.084
Observations	9		52		
2015–2020					
TFP	1.313	1.097	1.159	0.648	0.154
PEFF	1.078	0.382	1.051	0.540	0.027
TECH	1.121	0.332	1.085	0.330	0.036
SC	0.979	0.095	1.035	0.212	−0.056
Observations	40		376		

The table shows the summary statistics of hospitals’ TFP and its components for both groups of hospitals, in terms of whether or not VIP medical services are provided.

TECH, Technical; PEFF, Pure Efficiency; SC, Scale.

### Effect of VIP beds on hospital operating efficiency

VIP medical services and hospital TFP were linked in the second-stage regression. Appendix Tables 3, 4) show the baseline results for the two-way FE model and panel Tobit model, respectively. TFP, PEFF, TECH, and SC are the dependent variables. Because of their high correlations, TFP and its decomposition term yield similar results in many cases. After adding all control variables, regardless of which model or proxy variable is used, VIP medical services are found to have a significant negative impact on hospital TFP and some sub-indices (columns (1) (5) (9) in Appendix Tables 3, 4). Using whether VIP beds are available or not as the proxy variable, the decrease of TFP in hospitals is found to be primarily caused by the decrease of TECH and SC (columns (1) (3) (4) in Appendix Tables 3, 4). When using the ratio of VIP beds as the proxy variable, the decrease of TFP in hospitals primarily results from the decrease of PEFF and TECH (columns (5) (6) (7) in Appendix Tables 3, 4). When the number of VIP beds is the proxy variable, the decrease of TFP in hospitals is primarily caused by the decrease of SE (columns (9) (12) in Appendix Tables 3, 4). Simultaneously, a higher hospital ranking is also associated with lower TFP. Hospitals’ TFP is higher when doctors diagnose more patients on average.

Coefficients for the medical institution’s external characteristics such as ownership, specialization, profit or not, or capital intensity are occasionally significant but did not seem to have a systematic impact. The same is true for our socio-economic control variables (due to space constraints, detailed regression results for control variables in Appendix Tables 3, 4). Given that the Tobit approach is less biased when analyzing censored data, it is more reliable than the two-way FE model. However, the same conclusions were obtained from both the two-way FE model and the panel Tobit model. Our results indicate that VIP medical services are an important factor in the productivity of hospitals. As the baseline estimations can be tempered with the possibility of an endogeneity problem, the following section focuses on the results from the instrumental variable estimations.

### IV regression results

Table 3 reports the effect of the number of key departments in preparation on VIP beds available or not (columns (1) in Table 3). After adding environment and medical institution characteristics controls, the relationship is strong. The number of key departments in preparation has a statistically significant positive impact on whether VIP beds are available. The Kleibergen–Paap rk LM statistic, Cragg–Donald Wald F statistic and Kleibergen–Paap rk Wald F statistic in column (1) are well above the critical value (value in parentheses). This preferred estimation suggests that when the number of key departments in preparation increases by 1, whether the hospital has VIP beds increases by 0.103. As discussed in Section 2.3.2, the censor data calls for the use of corner solution models. We used two-step IV-Tobit estimations with the lower limit for left censoring at zero. The incidental parameter problem prohibits the application of individual fixed effects. Therefore, we replaced the measure of VIP medical services with the number of VIP beds instead of the dummy variable for whether VIP beds are available or not. The relationship between the IV variables (proportion of transportation land area and total hospital income) and the number of VIP beds remains statistically significantly positive.

**Table 3:** Regression results: effect of VIP beds on hospital operating efficiency (IV estimation).

	IV-Two-way FE					IV-Panel Tobit				
	First Stage	TFP	Thereof:			First Stage	TFP	Thereof:		
			PEFF	TECH	SC			PEFF	TECH	SC
	(1)	(2)	(3)	(4)	(5)	(6)	(7)	(8)	(9)	(10)
VIP beds available or not		−1.030***(0.384)	−0.260(0.218)	−0.324**(0.139)	−0.387***(0.130)					
Number of VIP beds							−0.035**(0.015)	−0.018*(0.011)	−0.006(0.006)	−0.006(0.004)
Number of key departments in preparation	0.103***(0.027)									
The proportion of transportation land area						154.313***(41.953)				
Total hospital income (10^8^ CNY, GDP deflated)						0.337***(0.038)				
Outside environment characteristics	YES	YES	YES	YES	YES	YES	YES	YES	YES	YES
Medical institution’s external characteristics	YES	YES	YES	YES	YES	YES	YES	YES	YES	YES
Medical institution policy-induced	YES	YES	YES	YES	YES	YES	YES	YES	YES	YES
Year fixed-effect	YES	YES	YES	YES	YES	YES	YES	YES	YES	YES
Individual fixed-effect	YES	YES	YES	YES	YES					
Kleibergen-Paap rk LM statistic	8.97***									
Cragg-Donald Wald F statistic	31.659* {16.38}									
Kleibergen-Paap rk Wald F statistic	14.518^#^ {8.96}									
Observations	416	416	416	416	416	416	416	416	416	416
R-Square		0.189	0.198	0.218	0.046					

Cluster standard errors in parentheses. ^#^*p* < 0.5,*p < 0.1, **p < 0.05, ***p < 0.01.

TECH, Technical; PEFF, Pure Efficiency; SC, Scale.

Table 3 presents the estimate of the effect of VIP medical services on hospital operating efficiency and its decomposition. IV-FE estimates are reported in columns (2)–(5), and IV-Tobit estimates are shown in columns (7)–(10). In all regressions, the environment and medical institution characteristics were under control. We found that in the IV estimation, VIP medical services had negative and statistically significant impacts on hospital efficiency. This shows that the opening or expanding of VIP medical services leads to the deterioration of hospital TFP. Specifically, when the number of VIP beds increases by 1, the TFP of the hospital decreases by 0.035. Similarly, if a hospital’s chances of providing VIP medical services increase by 10%, its TFP decreases by 0.0103, *ceteris paribus*. This finding is consistent with previous discussions regarding concierge medicine (3, 14, 59). The mechanisms will be discussed in detail later.

The IV estimation of the decomposition term was consistent with the basic regression; we found that the opening of VIP medical services had a significantly negative impact on TECH and SC (columnd (4) (5) in Table 3). This finding is contrary to popular perception. Instead of improving the technical efficiency of hospitals by promoting the use of high-end technology or advanced equipment, establishing VIP medical services decreases technical progress (TECH) and scale efficiency (SC). When this differentiated service for small groups reduces the overall efficiency of the scale, the technical efficiency also decreases, which is why the overall TFP decreases once the hospital provides VIP care. This is consistent with Romer’s Law, namely that the introduction of medical devices creates demand for medical devices and leads to an increase in procedures and programs for patient treatment and examination, thus affecting the technological progress of medical institutions. A similar situation has occurred in VIP medicine practice, where greater reliance on medical examination equipment compared to general medicine has hindered technological progress.

However, when the number of VIP beds increases, the effects of TECH and SC become insignificant; the decrease in PEFF was the main reason for the continued deterioration of TFP [column (8) in Table 3]. A possible explanation for this is the mismatch of resources caused by the expansion of the VIP medical scale. In practice, VIP medical care concentrates medical resources on a small number of people who buy VIP services rather than ensures an optimal match between supply and demand. Therefore, more experienced doctors will spend more time treating such patients and less time on general medical services due to financial incentives. Additionally, patients who receive VIP services may not be seriously ill, while seriously ill patients who need specialist treatment cannot receive priority care because of their poverty. This mismatch of resources affects the improvement of medical services’ efficiency (PEFF).

### Robustness check

Identifying causality relies on the selection of a control group. Based on the previous analysis, whether hospitals provide VIP services may not be completely random, which may cause self-selection bias. To alleviate this endogeneity, the non-alternative one-to-one nearest neighbor matching method (60, 61) was used to match the control group for each year’s treatment group based on propensity score matching (62). Combined with the observable matching variables (regional GDP *per capita* (GDP deflated), ownership, profit or not, and support health insurance or not), the predicted probability of each hospital offering VIP medical care was calculated, and then the only control group that did not provide VIP medical care was found for each hospital providing VIP medical services. Finally, 364 observations were obtained. The same analyses in Appendix Tables 3, 4 were repeated and the results are presented in Panel A of Appendix Table 6. Our results before and after matching are consistent with some minor differences. Hospitals with VIP medical services have worse TFP under the same control variable conditions as the baseline regression, and the impact remains statistically significant regardless of the estimation method or independent variable measure. However, the difference in regression coefficients before and after matching is larger for the two-way FE than the panel Tobit model. This may be because the Tobit model has greater advantages in addressing censored data.

Given the unpredictable impact of COVID-19 on hospital productivity, observations in 2020 were deleted and 355 observations that remained from 2015 to 2019 were analyzed. The estimates are reported in Panel B of Appendix Table 6. The significance of the regression coefficients estimated by Tobit decreased slightly, except that the number of VIP beds had no significant effect on TFP; the other coefficients were all statistically significant at the 10% level. In both specifications, the estimates were consistent with our baseline results. VIP services reduce hospital productivity in China. Similarly, we conducted a robustness test on the hypothesis that the base year TFP was 1. The observations in 2015 were deleted, and the remaining 342 observations from 2016 to 2020 were analyzed. These estimates are presented in Panel C of Appendix Table 6. The direction and significance of the regression coefficients of OLS or Tobit are consistent with the original regression (the absolute value of the OLS coefficient becomes smaller, and the Tobit coefficient becomes larger). The introduction and operation of VIP services in China reduces hospital productivity, and the robustness of this conclusion is not affected by the assumption that the base year TFP is 1.

To avoid the possibility of bias in index selection and to test the robustness of results in the estimation, quality-adjusted discharges were further introduced into hospital productivity valuation. Successful treatments and quality improvements in hospital care materialize as decreased hospital mortality (26, 63). The health improvement resulting from hospital care is in-hospital mortality subtracted from overall population mortality (e.g., if the in-hospital mortality rate decreases by 10% and the overall population mortality rate decreases by 5%, the hospital-related decrease in mortality amounts to 10–5% = 5%. The quality index increases from 100 to (100 × 1.05) = 105). We defined hospital outcome (quality-adjusted discharges) as the product of the number of discharges and the mortality-based quality index. With the same method, we obtained outpatient and emergency quality-adjusted discharges. The quality-adjusted discharges of outpatient and emergency care and hospitalization were included in the output indicators to obtain the medical institute TFP, which accounted for social benefits. The estimates are reported in Panel D of Appendix Table 6. The main results remains robust, suggesting that the TFP considering social benefit and the TFP considering only economic benefit do not affect the significance of the regression results. In other words, opening VIP medical treatment negatively affects the operating efficiency of hospitals.

In addition, traditional split DEA two-stage method lacks a clear theory of the basic data generation process and ignores the fact that the estimated DEA efficiency score is calculated based on common data samples. Treating them as independent observations invalidates any inferences because they are related to the sequence (64). Following Badunenko and Tauchmann (65), we re-performed the two-stage efficiency analysis in Stata16.0 using the command *simarwilson* combined with the bootstrapped parameter program and used this result as a robustness check (Appendix Table 6–2). The analysis results are consistent with the results of the baseline regression OLS/Tobit (although there are numerical differences, the direction and significance are consistent).

Finally, considering that we used unbalanced panel data, missing values may have caused bias in TFP measurement. Therefore, we processed the original data into balanced panel data, removed medical institutions with missing values during the sample observation period, and finally calculated 375 TFP values of 75 medical institutions and included them in the second-stage regression analysis. As above, we used the two-way FE model, Panel-Tobit model, and *simarwilson* estimation (2000 bootstrap reps) as robustness tests to explore the impact of VIP medical services on the TFP of medical institutions (Appendix Table 6–3). The final regression results are consistent with the results obtained using unbalanced panel data; the direction and significance of the regression coefficients are largely consistent, and the absolute values are relatively small.

### Effect heterogeneity

Appendix Table 7 presents the impacts of VIP medical hospital TFP across sub-groups. According to the average regional GDP, the observations were divided into groups with high and low economic development. We found that VIP medical services had a greater impact among the high economic development group, and the negative effect of VIP medical services on TFP was statistically significant in this group (columns (1)–(12) in Panel A of Appendix Table 7). It was also consistent with the subgroup analysis based on health resource abundance (regions with higher economic levels tend to have more adequate medical resources). Once hospitals with more advantageous resource endowments provide VIP services, they tend to have poorer productivity performance (columns (1)–(12) in Panel B of Appendix Table 7). This finding may be explained by the same mechanism. In areas with better economic conditions or more health resources, when hospitals provide VIP medical care, resource mismatch between patients and doctors will occur more readily due to the presence of more high-end medical consumers. Additionally, medical institutions in these areas may be more crowded and difficult to manage as better resources lead to a large influx of patients. This situation will affect the hospital’s management efficiency and lead to the further deterioration of TFP. Further, hospitals that are part of groups with better medical resources/financial status are more likely to have more advanced examination technology and equipment, and this is likely to result in the overuse of technology and hinder the technological progress of the hospitals, resulting in a further negative impact on TFP.

We also examined the heterogeneity of ownership using different VIP medical service proxy variables and estimation methods (Panel C of Appendix Table 7). According to the results, there was no significant heterogeneity in the opening of VIP services on TFP (columns (1), (4), (7), and (10) in Panel B of Appendix Table 7). In terms of VIP medical beds, ownership plays a role in efficiency performance, as we found in the Deyang sample. There exists robust evidence that the increase of VIP medical beds in private hospitals has a more serious negative impact on the overall TFP of hospitals (columns (8) and (11) in Panel B of Appendix Table 7). This may be because private hospitals are more obviously profit-oriented than public hospitals. The significant financial incentive caused by the medical supply mode of VIP medical treatment is more likely to lead to management chaos and affect the TFP of hospitals. Regarding the heterogeneity of different specializations (columns (1)–(12) in Panel D of Appendix Table 7), the impact of VIP medical services on hospital efficiency exhibited differences. The negative impact on the efficiency of medical institutions was more profound in specialized hospitals, whether they were setting up VIP medical services or expanding their scale. This may be because specialized hospitals are predominately comprised of departments with a strong dependence on technology or products (e.g., oncology, stomatology, cosmetology), and serving special medical needs may promote the over-utilization of technology, which affects technological progress and overall TFP.

## Discussion and conclusion

This study explored the causal relationship between VIP medical services and hospital efficiency in China. Using the number of key departments in preparation as the instrumental variable, we found that opening VIP medical services led to the deterioration of a hospital’s TFP. Specifically, when the number of VIP beds increases by 1, the TFP of the hospital decreases by 0.035. Similarly, if a hospital’s chance of providing VIP medical services increases by 10%, its TFP decreases by 0.0103, *ceteris paribus*. Our results were robust to alternative measures of VIP medical services and estimation methods.

By examining the medical service institutions in Deyang, our findings provide empirical evidence for the current development of VIP medical services in China. Contrary to the expectation of satisfying patients’ diverse medical services demands and improving doctors’ financial incentives to liberate the productivity of the medical institutions, the analysis of TFP and its decomposition items showed that overall hospital TFP was worsened by the provision of VIP medical services through two main channels. First, VIP medical treatment leads to a mismatch between doctors and patients, reduces resource efficiency, affects management efficiency, and damages the pure technical efficiency of medical services. This is consistent with previous research on VIP medicine affecting health care allocation, with those living in wealthier areas more likely to benefit from concierge medicine, while those living in less affluent areas experience interruptions in care (16). Since its supply and demand are regulated by market prices, VIP medicine does not divert more time and attention to patients in worse health but leads to more resources concentrated in the hands of the rich (66). From the perspective of medical services consumers, two types of individuals selected VIP medical services. One is the release of medical demand in the form of VIP medical care (67), and the other is a moral hazard, in that such services may needlessly waste too many medical resources. The latter issue might drive an even larger wedge between the marginal benefits and costs of care (68), which will undoubtedly aggravate the deterioration of hospital efficiency. Additionally, part of the service time of general medical care is occupied by special medical care, which aggravates the crowding of the resource-constrained medical market and ultimately affects the management efficiency and pure technical efficiency of the hospital overall. In addition, financial incentives for VIP care make hospitals more dependent on technology and equipment, which impedes the technical progress of medical workers. The “VIP syndrome” (29) may influence doctors’ medical decisions, and owing to their defensive medical motivation (15, 23), doctors may rely on examination equipment more than disease-based needs, which hinders doctors’ “learning by doing.”

Diverse medical demand has given rise to differentiated medical service forms and projects (4, 69, 70). However, for regions or countries with relatively insufficient and unbalanced medical resources, this research concludes that the service forms of VIP medical care will harm hospital TFP, and policy restrictions on VIP medical care are still necessary. Moreover, this effect is heterogeneous across groups, divided by the nature of the hospital and its environment. The better the economic development and the richer the medical resources, the more serious the deterioration of TFP caused by providing or expanding the scale of the VIP services. In the samples of private and specialized hospitals, the supply of VIP medical services had greater negative impact on TFP relative than other groups. These results indicate that within the Chinese context, establishing VIP medical services is not conducive to the performance of hospitals. This is consistent with previous studies that suggest that the “trickle-down effect” in the medical services may not be realistic (23), because the service form of VIP care worsens the efficiency of institutions in public hospitals with more medical resources or in specialized hospitals with more expertise in the field. Further, these hospitals, which correspond to higher profits, are not associated with higher levels of unpaid or “charitable” care (23). A study evaluating the provision of charitable care in non-profit hospitals in the United States found that hospitals with higher net income provided much less charitable care than hospitals with lower net income (71). Therefore, VIP revenues do not trickle down to disadvantaged groups.

Therefore, for some medical service institutions that do not carry out VIP care at present, the attitude toward VIP services should be more cautious, especially for those specialized and private hospitals with better economic resources and medical resources endowment. However, for hospitals that already provide VIP care, it is worth implementing more optimized process management to guide the proper matching of patients and doctors instead of relying on the price regulation of VIP services, strengthening the training of clinical and scientific research capabilities of medical personnel, guiding doctors to rationally use medical auxiliary equipment and examination under the medical guidelines, and standardizing the overall hospital diagnosis and treatment process. Additionally, in evaluating how VIP care fits into one country’s health care system, health equity should be used as the moral compass for creating more ethical systems.

This study has some substantial limitations. First, as the data were sourced from a prefecture-level city in western China, the research sample may lack generalisability, and our findings may not be directly applicable to different national contexts. For example, in developed countries, factors such as the medical culture, patient triage systems in medical institutions, and the allocation of medical resources may differ from the practice of VIP medical care in China. Therefore, the impacts of VIP medical projects on hospital TFP may vary. Second, due to the limitation of data availability, the use of unbalanced panel data to measure TFP may result in bias, or the economic significance of the results may be difficult to explain. If balanced panel data with a larger sample size are available, the accuracy of TFP measurement can be improved. Otherwise, the semi-parametric LP method, stochastic frontier production function and Cobb–Douglas production function can be used in a follow-up study to process unbalanced panel data to increase the accuracy of TFP measurement. Future research should also expand on our study by incorporating other techniques and hypotheses. For example, regarding TFP measurement and decomposition, estimation techniques such as two-stage semi-parametric models, bootstrapped double-frontier DEA, or network DEA (64, 72–76) in the first stage, and truncated regression, artificial intelligence, or machine learning (77–79) in the second stage can improve the robustness and predictive power of the model. Additionally, more precise environmental policy variables and longer datasets can also be used to strengthen our findings; these may be useful, for example, in determining whether there exists a U-shaped relationship between VIP healthcare and TFP. These aspects should be considered in future research.

## Data availability statement

The datasets presented in this article are not readily available because the data are not publicly available due to privacy or ethical restrictions. Requests to access the datasets should be directed to YY, yangyan_cau@163.com.

## Author contributions

YY: Data curation, Formal analysis, Methodology, Software, Writing – original draft, Writing – review & editing. MC: Conceptualization, Project administration, Supervision, Funding acquisition, Writing – original draft, Writing – review & editing. NC: Formal analysis, Methodology, Writing – review & editing. LY: Formal analysis, Methodology, Writing – review & editing. ZW: Investigation, Supervision, Resources, Validation, Visualization, Funding acquisition, Writing – original draft, Writing – review & editing.
