# Supplementary material for: Do VIP medical services damage efficiency? New evidence of medical institutions’ total factor productivity using Chinese panel data
Source: Front Public Health. 2024 Jan 24;11:1261804. doi: 10.3389/fpubh.2023.1261804 (PMC10847260; doi:10.3389/fpubh.2023.1261804)
Supplement: Supplementary file 1 [file Data_Sheet_1.doc]

Appendix

**Table 3 Regression results of VIP beds on hospital operating efficiency (Two-way FE model)**

|  | TFP | Thereof: | | | TFP | Thereof: | | | TFP | Thereof: | | |
| --- | --- | --- | --- | --- | --- | --- | --- | --- | --- | --- | --- | --- |
|  |  | PEFF | TECH | SC |  | PEFF | TECH | SC |  | PEFF | TECH | SC |
|  | (1) | (2) | (3) | (4) | (5) | (6) | (7) | (8) | (9) | (10) | (11) | (12) |
| VIP beds available or not | -0.318*** | -0.083 | -0.151** | -0.060** |  |  |  |  |  |  |  |  |
|  | (0.103) | (0.060) | (0.060) | (0.026) |  |  |  |  |  |  |  |  |
| Ratio of VIP beds |  |  |  |  | -4.654*** | -1.628** | -2.095*** | -0.540 |  |  |  |  |
|  |  |  |  |  | (1.419) | (0.813) | (0.679) | (0.365) |  |  |  |  |
| Ln Number of VIP beds |  |  |  |  |  |  |  |  | -0.117** | -0.033 | -0.053** | -0.018* |
|  |  |  |  |  |  |  |  |  | (0.045) | (0.022) | (0.023) | (0.010) |
| **Outside environment characteristics** | |  |  |  |  |  |  |  |  |  |  |  |
| Ln Regional GDP per capital (GDP deflated) | 1.007 | 0.820 | -0.017 | 0.240* | 1.070 | 0.833 | 0.014 | 0.254* | 0.999 | 0.815 | -0.018 | 0.241* |
|  | (0.675) | (0.611) | (0.180) | (0.135) | (0.671) | (0.608) | (0.184) | (0.139) | (0.676) | (0.613) | (0.180) | (0.136) |
| Ln Population density | 11.602 | 7.077 | 3.816 | 1.674 | 11.280 | 6.976 | 3.669 | 1.628 | 11.273 | 6.987 | 3.665 | 1.619 |
|  | (7.066) | (4.619) | (3.884) | (2.221) | (7.121) | (4.608) | (3.899) | (2.229) | (7.086) | (4.595) | (3.891) | (2.222) |
| Ln Urban unemployment rate | -7.626*** | -4.094* | -2.487** | -0.137 | -7.369*** | -4.052* | -2.359** | -0.069 | -7.537*** | -4.081* | -2.435** | -0.106 |
|  | (2.780) | (2.353) | (0.995) | (0.542) | (2.740) | (2.312) | (0.974) | (0.536) | (2.776) | (2.341) | (0.992) | (0.540) |
| Ln Population mortality | 0.054 | 0.091 | -0.002 | 0.018 | 0.053 | 0.091 | -0.003 | 0.018 | 0.046 | 0.089 | -0.006 | 0.017 |
|  | (0.146) | (0.119) | (0.067) | (0.024) | (0.146) | (0.119) | (0.066) | (0.023) | (0.146) | (0.119) | (0.066) | (0.024) |
| Ln Government attention to health | -0.452** | -0.211 | -0.161** | -0.025 | -0.433** | -0.208 | -0.152** | -0.020 | -0.450** | -0.211 | -0.160** | -0.024 |
|  | (0.215) | (0.180) | (0.065) | (0.035) | (0.212) | (0.177) | (0.063) | (0.034) | (0.216) | (0.180) | (0.065) | (0.035) |
| **Medical institution external characteristics** | | |  |  |  |  |  |  |  |  |  |  |
| Ln Governmental subsidy | 0.101 | 0.188 | 0.089 | -0.024 | 0.073 | 0.183 | 0.075 | -0.031 | 0.129 | 0.197 | 0.100 | -0.022 |
|  | (0.505) | (0.295) | (0.227) | (0.104) | (0.506) | (0.298) | (0.225) | (0.103) | (0.504) | (0.294) | (0.229) | (0.104) |
| Ownership | 0.173 | 0.203** | -0.043 | -0.024 | 0.174 | 0.204** | -0.043 | -0.024 | 0.174 | 0.204** | -0.043 | -0.024 |
|  | (0.183) | (0.099) | (0.068) | (0.046) | (0.182) | (0.099) | (0.067) | (0.045) | (0.183) | (0.099) | (0.068) | (0.045) |
| Specialization | 0.010 | 0.003 | -0.011 | 0.046 | 0.011 | 0.004 | -0.011 | 0.045 | 0.008 | 0.002 | -0.012 | 0.045 |
|  | (0.160) | (0.101) | (0.049) | (0.041) | (0.159) | (0.101) | (0.048) | (0.041) | (0.159) | (0.101) | (0.049) | (0.041) |
| Profit or not | -0.028 | -0.002 | -0.020 | 0.015 | -0.022 | -0.001 | -0.017 | 0.017 | -0.026 | -0.001 | -0.018 | 0.016 |
|  | (0.168) | (0.081) | (0.069) | (0.046) | (0.166) | (0.081) | (0.069) | (0.046) | (0.168) | (0.081) | (0.069) | (0.046) |
| Medical students training base or not | 0.067 | -0.034 | 0.023 | 0.033 | 0.027 | -0.046 | 0.004 | 0.027 | 0.055 | -0.037 | 0.017 | 0.031 |
|  | (0.070) | (0.059) | (0.046) | (0.028) | (0.076) | (0.059) | (0.037) | (0.033) | (0.067) | (0.058) | (0.045) | (0.028) |
| Support health insurance or not | 0.163 | 0.277*** | -0.149 | -0.030 | 0.161 | 0.277*** | -0.151 | -0.032 | 0.161 | 0.277*** | -0.151 | -0.031 |
|  | (0.208) | (0.100) | (0.183) | (0.039) | (0.211) | (0.100) | (0.185) | (0.039) | (0.209) | (0.100) | (0.184) | (0.039) |
| Rank | -0.179* | -0.154 | 0.005 | -0.018 | -0.183* | -0.155 | 0.003 | -0.019 | -0.184* | -0.155 | 0.003 | -0.019 |
|  | (0.104) | (0.107) | (0.038) | (0.026) | (0.103) | (0.107) | (0.038) | (0.026) | (0.103) | (0.107) | (0.037) | (0.026) |
| Ln Capital intensity | -0.104 | 0.059 | -0.114** | -0.026 | -0.103 | 0.058 | -0.114** | -0.025 | -0.101 | 0.059 | -0.112** | -0.025 |
|  | (0.102) | (0.050) | (0.046) | (0.025) | (0.102) | (0.050) | (0.047) | (0.025) | (0.102) | (0.050) | (0.046) | (0.025) |
| Ln Number of hospital beds | -0.074 | -0.214 | 0.064 | 0.062 | -0.071 | -0.215 | 0.066 | 0.063 | -0.069 | -0.214 | 0.066 | 0.063 |
|  | (0.267) | (0.240) | (0.081) | (0.068) | (0.266) | (0.240) | (0.081) | (0.068) | (0.267) | (0.240) | (0.081) | (0.068) |
| Ln Number of key departments | -0.157 | 0.014 | -0.048 | -0.057 | -0.173 | 0.013 | -0.057 | -0.062 | -0.143 | 0.019 | -0.043 | -0.056 |
|  | (0.109) | (0.070) | (0.039) | (0.051) | (0.122) | (0.067) | (0.044) | (0.053) | (0.117) | (0.070) | (0.041) | (0.053) |
| Ln Equipment value above 10,000 yuan (GDP deflated) | -0.010 | 0.015 | -0.015 | 0.004 | -0.010 | 0.016 | -0.014 | 0.004 | -0.012 | 0.015 | -0.015 | 0.004 |
|  | (0.030) | (0.016) | (0.020) | (0.008) | (0.030) | (0.016) | (0.020) | (0.008) | (0.030) | (0.016) | (0.020) | (0.008) |
| Ln Average outpatient cost (GDP deflated) | 0.101 | -0.018 | 0.058 | 0.001 | 0.101 | -0.019 | 0.058 | 0.002 | 0.103 | -0.018 | 0.059 | 0.002 |
|  | (0.139) | (0.072) | (0.050) | (0.028) | (0.140) | (0.072) | (0.050) | (0.028) | (0.140) | (0.072) | (0.050) | (0.028) |
| **Medical institution policy induced** | |  |  |  |  |  |  |  |  |  |  |  |
| Ratio of nurses to physicians | 0.101 | -0.018 | 0.058 | 0.001 | 0.101 | -0.019 | 0.058 | 0.002 | 0.728*** | 0.365** | 0.154 | 0.093 |
|  | (0.139) | (0.072) | (0.050) | (0.028) | (0.140) | (0.072) | (0.050) | (0.028) | (0.246) | (0.166) | (0.110) | (0.060) |
| Ratio of beds to nurses | -0.056 | 0.020 | -0.038** | -0.012 | -0.056 | 0.020 | -0.037** | -0.012 | -0.056 | 0.020 | -0.037** | -0.012 |
|  | (0.062) | (0.046) | (0.019) | (0.014) | (0.062) | (0.047) | (0.019) | (0.014) | (0.061) | (0.046) | (0.019) | (0.014) |
| Ln Beds occupancy rate | 0.000 | 0.050* | 0.006 | -0.082* | -0.002 | 0.050* | 0.005 | -0.083* | -0.002 | 0.050* | 0.005 | -0.083* |
|  | (0.053) | (0.030) | (0.032) | (0.049) | (0.053) | (0.030) | (0.032) | (0.049) | (0.053) | (0.030) | (0.032) | (0.049) |
| Ln Average length of stay for discharged patients | 0.131 | -0.022 | 0.110 | -0.007 | 0.124 | -0.024 | 0.107 | -0.009 | 0.126 | -0.023 | 0.108 | -0.008 |
|  | (0.118) | (0.068) | (0.101) | (0.021) | (0.118) | (0.068) | (0.101) | (0.021) | (0.118) | (0.068) | (0.101) | (0.021) |
| Ln Average number of patients physicians diagnosed | 0.482*** | 0.328*** | 0.095 | 0.038 | 0.479*** | 0.327*** | 0.093 | 0.037 | 0.482*** | 0.328*** | 0.094 | 0.038 |
|  | (0.154) | (0.119) | (0.063) | (0.038) | (0.155) | (0.120) | (0.063) | (0.038) | (0.155) | (0.119) | (0.063) | (0.038) |
| Constant | -79.493* | -51.193* | -21.251 | -12.555 | -78.403* | -50.712* | -20.787 | -12.509 | -77.351* | -50.561* | -20.308 | -12.257 |
|  | (46.069) | (30.282) | (26.090) | (14.929) | (46.605) | (30.290) | (26.316) | (15.026) | (46.321) | (30.182) | (26.179) | (14.970) |
| Year fixed effect | YES | YES | YES | YES | YES | YES | YES | YES | YES | YES | YES | YES |
| Individual fixed effect | YES | YES | YES | YES | YES | YES | YES | YES | YES | YES | YES | YES |
| Observations | 416 | 416 | 416 | 416 | 416 | 416 | 416 | 416 | 416 | 416 | 416 | 416 |
| Adjusted R-Square | 0.236 | 0.204 | 0.231 | 0.199 | 0.233 | 0.204 | 0.227 | 0.195 | 0.234 | 0.204 | 0.228 | 0.196 |

Cluster standard errors in parentheses

* p<0.1, ** p<0.05, *** p<0.01

TECH: Technical; PEFF: Pure Efficiency; SC: Scale.

**Table 4 Regression results of VIP beds on hospital operating efficiency (Panel Tobit model)**

|  | TFP | Thereof: | | | TFP | Thereof: | | | TFP | Thereof: | | |
| --- | --- | --- | --- | --- | --- | --- | --- | --- | --- | --- | --- | --- |
|  |  | PEFF | TECH | SC |  | PEFF | TECH | SC |  | PEFF | TECH | SC |
|  | (1) | (2) | (3) | (4) | (5) | (6) | (7) | (8) | (9) | (10) | (11) | (12) |
| VIP beds available or not | -0.126*** | -0.007 | -0.014** | -0.021*** |  |  |  |  |  |  |  |  |
|  | (0.047) | (0.022) | (0.007) | (0.003) |  |  |  |  |  |  |  |  |
| Ratio of VIP beds |  |  |  |  | -3.209*** | -0.949** | -0.347*** | -0.288 |  |  |  |  |
|  |  |  |  |  | (0.599) | (0.412) | (0.032) | (0.324) |  |  |  |  |
| Ln Number of VIP beds |  |  |  |  |  |  |  |  | -0.031*** | 0.005 | -0.002 | -0.007*** |
|  |  |  |  |  |  |  |  |  | (0.012) | (0.006) | (0.004) | (0.002) |
| **Outside environment characteristics** | |  |  |  |  |  |  |  |  |  |  |  |
| Ln Regional GDP per capital (GDP deflated) | 0.207*** | 0.004 | 0.146*** | -0.013 | 0.215*** | 0.020 | 0.146*** | -0.014 | 0.199*** | -0.001 | 0.144*** | -0.013 |
|  | (0.070) | (0.024) | (0.020) | (0.010) | (0.067) | (0.025) | (0.021) | (0.010) | (0.065) | (0.024) | (0.019) | (0.010) |
| Ln Population density | -0.134*** | 0.066*** | -0.178*** | -0.020** | -0.140*** | 0.061*** | -0.178*** | -0.021* | -0.136*** | 0.068*** | -0.179*** | -0.021** |
|  | (0.050) | (0.004) | (0.012) | (0.010) | (0.050) | (0.005) | (0.013) | (0.011) | (0.052) | (0.006) | (0.012) | (0.010) |
| Ln Urban unemployment rate | 0.011 | 0.010 | 0.002 | 0.000 | 0.012 | 0.010 | 0.002 | 0.000 | 0.012 | 0.010 | 0.002 | 0.000 |
|  | (.) | (.) | (.) | (.) | (.) | (.) | (.) | (.) | (.) | (.) | (.) | (.) |
| Ln Population mortality | -0.093** | -0.128*** | 0.005 | 0.013 | -0.102*** | -0.140*** | 0.005 | 0.013 | -0.090** | -0.127*** | 0.006 | 0.013 |
|  | (0.037) | (0.025) | (0.005) | (0.010) | (0.035) | (0.027) | (0.004) | (0.013) | (0.039) | (0.026) | (0.006) | (0.011) |
| Ln Government attention to health | 1.368 | 1.285 | -0.118 | 0.507 | 1.246 | 1.242 | -0.142 | 0.514 | 1.345 | 1.293 | -0.124 | 0.508 |
|  | (.) | (.) | (.) | (.) | (.) | (.) | (.) | (.) | (.) | (.) | (.) | (.) |
| **Medical institution external characteristics** | | |  |  |  |  |  |  |  |  |  |  |
| Ln Governmental subsidy | 0.498*** | 0.074 | 0.282** | 0.086*** | 0.542*** | 0.114 | 0.284** | 0.088*** | 0.484*** | 0.070 | 0.283** | 0.083*** |
|  | (0.178) | (0.100) | (0.111) | (0.022) | (0.177) | (0.108) | (0.114) | (0.012) | (0.164) | (0.091) | (0.115) | (0.019) |
| Ownership | -0.087** | -0.034* | -0.093*** | -0.025*** | -0.085** | -0.030 | -0.093*** | -0.024*** | -0.092*** | -0.036** | -0.094*** | -0.025*** |
|  | (0.037) | (0.018) | (0.010) | (0.005) | (0.038) | (0.021) | (0.010) | (0.006) | (0.030) | (0.018) | (0.011) | (0.005) |
| Specialization | 0.064*** | -0.049*** | 0.042*** | 0.011** | 0.066*** | -0.044*** | 0.042*** | 0.012** | 0.059*** | -0.050*** | 0.042*** | 0.011** |
|  | (0.024) | (0.002) | (0.003) | (0.005) | (0.024) | (0.003) | (0.003) | (0.006) | (0.021) | (0.003) | (0.003) | (0.005) |
| Profit or not | 0.103* | 0.007 | -0.008 | 0.008 | 0.113* | 0.009 | -0.007 | 0.010 | 0.103* | 0.008 | -0.007 | 0.009 |
|  | (0.056) | (0.018) | (0.005) | (0.007) | (0.060) | (0.019) | (0.006) | (0.007) | (0.055) | (0.019) | (0.005) | (0.007) |
| Medical students training base or not | -0.029 | -0.036*** | 0.012 | -0.004 | -0.036 | -0.032*** | 0.010 | -0.006*** | -0.033 | -0.041*** | 0.010 | -0.002 |
|  | (0.044) | (0.006) | (0.018) | (0.010) | (0.028) | (0.007) | (0.021) | (0.002) | (0.037) | (0.011) | (0.015) | (0.006) |
| Support health insurance or not | -0.048 | 0.115* | -0.110 | -0.037 | -0.042 | 0.111* | -0.112 | -0.035 | -0.047 | 0.121** | -0.109 | -0.037 |
|  | (0.068) | (0.063) | (0.244) | (0.036) | (0.078) | (0.060) | (0.242) | (0.040) | (0.069) | (0.062) | (0.243) | (0.033) |
| Rank | -0.043* | -0.006 | -0.015 | -0.002 | -0.048* | -0.008 | -0.015 | -0.003 | -0.045* | -0.005 | -0.015 | -0.002 |
|  | (0.025) | (0.008) | (0.010) | (0.005) | (0.027) | (0.008) | (0.010) | (0.006) | (0.026) | (0.009) | (0.010) | (0.005) |
| Ln Capital intensity | 0.022*** | 0.069*** | -0.021*** | -0.006** | 0.022*** | 0.066*** | -0.021*** | -0.005** | 0.024*** | 0.070*** | -0.021*** | -0.006** |
|  | (0.002) | (0.003) | (0.001) | (0.003) | (0.001) | (0.003) | (0.001) | (0.002) | (0.001) | (0.004) | (0.001) | (0.003) |
| Ln Number of hospital beds | 0.022*** | -0.064*** | 0.040*** | 0.018*** | 0.025*** | -0.059*** | 0.041*** | 0.019*** | 0.021*** | -0.065*** | 0.040*** | 0.019*** |
|  | (0.000) | (0.002) | (0.006) | (0.003) | (0.002) | (0.003) | (0.006) | (0.002) | (0.001) | (0.002) | (0.006) | (0.003) |
| Ln Number of key departments | 0.059*** | 0.043*** | -0.025*** | 0.016*** | 0.061*** | 0.042*** | -0.025*** | 0.015*** | 0.065*** | 0.042*** | -0.025*** | 0.016*** |
|  | (0.003) | (0.002) | (0.008) | (0.003) | (0.001) | (0.003) | (0.008) | (0.004) | (0.001) | (0.001) | (0.007) | (0.003) |
| Ln Equipment value above 10,000 yuan (GDP deflated) | -0.001 | 0.011*** | -0.006*** | -0.000 | -0.002 | 0.013*** | -0.006** | -0.000 | -0.003 | 0.011*** | -0.007*** | -0.000 |
|  | (0.003) | (0.002) | (0.002) | (0.000) | (0.003) | (0.002) | (0.003) | (0.000) | (0.002) | (0.001) | (0.002) | (0.000) |
| Ln Average outpatient cost (GDP deflated) | 0.121*** | 0.075*** | 0.062*** | -0.010*** | 0.119*** | 0.074*** | 0.061*** | -0.010*** | 0.123*** | 0.075*** | 0.062*** | -0.009*** |
|  | (0.033) | (0.018) | (0.008) | (0.001) | (0.034) | (0.018) | (0.008) | (0.001) | (0.035) | (0.018) | (0.008) | (0.001) |
| **Medical institution policy induced** | |  |  |  |  |  |  |  |  |  |  |  |
| Ratio of nurses to physicians | -0.042** | -0.049*** | -0.019 | 0.005 | -0.050** | -0.052*** | -0.020 | 0.005 | -0.041** | -0.047*** | -0.019 | 0.005* |
|  | (0.020) | (0.007) | (0.026) | (0.004) | (0.025) | (0.008) | (0.026) | (0.003) | (0.020) | (0.006) | (0.025) | (0.003) |
| Ratio of beds to nurses | 0.013*** | 0.028*** | -0.012*** | -0.003*** | 0.014*** | 0.028*** | -0.012*** | -0.003*** | 0.012*** | 0.027*** | -0.012*** | -0.003*** |
|  | (0.003) | (0.000) | (0.003) | (0.001) | (0.004) | (0.000) | (0.003) | (0.001) | (0.002) | (0.000) | (0.003) | (0.001) |
| Ln Beds occupancy rate | 0.034** | 0.034*** | 0.018*** | -0.009** | 0.032** | 0.033*** | 0.018*** | -0.009*** | 0.033*** | 0.034*** | 0.018*** | -0.009** |
|  | (0.013) | (0.011) | (0.005) | (0.004) | (0.013) | (0.010) | (0.005) | (0.004) | (0.013) | (0.011) | (0.005) | (0.004) |
| Ln Average length of stay for discharged patients | -0.088*** | -0.043*** | 0.012 | -0.011*** | -0.094*** | -0.051*** | 0.012 | -0.011*** | -0.081*** | -0.041*** | 0.013 | -0.011*** |
|  | (0.015) | (0.009) | (0.012) | (0.001) | (0.016) | (0.009) | (0.011) | (0.002) | (0.012) | (0.008) | (0.012) | (0.001) |
| Ln Average number of patients physicians diagnosed | 0.056*** | 0.062*** | 0.006*** | 0.003*** | 0.056*** | 0.063*** | 0.006*** | 0.003*** | 0.054*** | 0.061*** | 0.005*** | 0.003*** |
|  | (0.015) | (0.008) | (0.000) | (0.001) | (0.015) | (0.008) | (0.000) | (0.000) | (0.015) | (0.008) | (0.000) | (0.001) |
| Year fixed effect | YES | YES | YES | YES | YES | YES | YES | YES | YES | YES | YES | YES |
| Individual fixed effect | YES | YES | YES | YES | YES | YES | YES | YES | YES | YES | YES | YES |
| Observations | 416 | 416 | 416 | 416 | 416 | 416 | 416 | 416 | 416 | 416 | 416 | 416 |

Cluster standard errors in parentheses

# p<0.015, * p<0.1, ** p<0.05, *** p<0.01

TECH: Technical; PEFF: Pure Efficiency; SC: Scale.

**Table 6 Robust check**

|  | TFP | | | | | |
| --- | --- | --- | --- | --- | --- | --- |
|  | Two-way FE | | | Panel Tobit model | | |
| **Panel A (After PSM)** | (1) | (2) | (3) | (4) | (5) | (6) |
| VIP beds available or not | -0.469*** |  |  | -0.118** |  |  |
|  | (0.137) |  |  | (0.048) |  |  |
| Ratio of VIP beds |  | -6.877*** |  |  | -3.279*** |  |
|  |  | (2.350) |  |  | (0.623) |  |
| Ln Number of VIP beds |  |  | -0.207** |  |  | -0.027** |
|  |  |  | (0.087) |  |  | (0.013) |
| Outside environment characteristics | YES | YES | YES | YES | YES | YES |
| Medical institution external characteristics | YES | YES | YES | YES | YES | YES |
| Medical institution policy induced | YES | YES | YES | YES | YES | YES |
| Year fixed effect | YES | YES | YES | YES | YES | YES |
| Individual fixed effect | YES | YES | YES | YES | YES | YES |
| Observations | 364 | 364 | 364 | 364 | 364 | 364 |
| R-Square | 0.260 | 0.254 | 0.258 |  |  |  |
| **Panel B (Excluding 2020 data)** | (7) | (8) | (9) | (10) | (11) | (12) |
| VIP beds available or not | -0.310*** |  |  | -0.084* |  |  |
|  | (0.106) |  |  | (0.048) |  |  |
| Ratio of VIP beds |  | -4.813*** |  |  | -2.547*** |  |
|  |  | (1.477) |  |  | (0.972) |  |
| Ln Number of VIP beds |  |  | -0.113** |  |  | -0.020 |
|  |  |  | (0.048) |  |  | (0.013) |
| Outside environment characteristics | YES | YES | YES | YES | YES | YES |
| Medical institution external characteristics | YES | YES | YES | YES | YES | YES |
| Medical institution policy induced | YES | YES | YES | YES | YES | YES |
| Year fixed effect | YES | YES | YES | YES | YES | YES |
| Individual fixed effect | YES | YES | YES | YES | YES | YES |
| Observations | 355 | 355 | 355 | 355 | 355 | 355 |
| R-Square | 0.255 | 0.252 | 0.252 |  |  |  |
| **Panel C (Excluding 2015 data)** | (13) | (14) | (15) | (16) | (17) | (18) |
| VIP beds available or not | -0.275** |  |  | -0.166*** |  |  |
|  | (0.106) |  |  | (0.054) |  |  |
| Ratio of VIP beds |  | -2.816** |  |  | -3.997*** |  |
|  |  | (1.076) |  |  | (0.469) |  |
| Ln Number of VIP beds |  |  | -0.098** |  |  | -0.040*** |
|  |  |  | (0.041) |  |  | (0.013) |
| Outside environment characteristics | YES | YES | YES | YES | YES | YES |
| Medical institution external characteristics | YES | YES | YES | YES | YES | YES |
| Medical institution policy induced | YES | YES | YES | YES | YES | YES |
| Year fixed effect | YES | YES | YES | YES | YES | YES |
| Individual fixed effect | YES | YES | YES | YES | YES | YES |
| Observations | 342 | 342 | 342 |  |  |  |
| R-Square | 0.208 | 0.203 | 0.296 |  |  |  |
| **Panel D (Exchange Output)** | (19) | (20) | (21) | (22) | (23) | (24) |
| VIP beds available or not | -0.308** |  |  | -0.115* |  |  |
|  | (0.117) |  |  | (0.064) |  |  |
| Ratio of VIP beds |  | -4.226*** |  |  | -2.866*** |  |
|  |  | (1.464) |  |  | (0.534) |  |
| Ln Number of VIP beds |  |  | -0.105** |  |  | -0.024 |
|  |  |  | (0.051) |  |  | (0.024) |
| Outside environment characteristics | YES | YES | YES | YES | YES | YES |
| Medical institution external characteristics | YES | YES | YES | YES | YES | YES |
| Medical institution policy induced | YES | YES | YES | YES | YES | YES |
| Year fixed effect | YES | YES | YES | YES | YES | YES |
| Individual fixed effect | YES | YES | YES | YES | YES | YES |
| Observations | 401 | 401 | 401 | 401 | 401 | 401 |
| R-Square | 0.266 | 0.261 | 0.262 |  |  |  |

Note: Cluster standard errors in parentheses* p<0.1, ** p<0.05, *** p<0.01

**Table 6-2 Robust check, Simarwilson Estimation results (Bootstrapped 2000 reps)**

|  | TFP | Thereof: | | | TFP | Thereof: | | | TFP | Thereof: | | |
| --- | --- | --- | --- | --- | --- | --- | --- | --- | --- | --- | --- | --- |
|  |  | PEFF | TECH | SC |  | PEFF | TECH | SC |  | PEFF | TECH | SC |
|  | (1) | (2) | (3) | (4) | (5) | (6) | (7) | (8) | (9) | (10) | (11) | (12) |
| VIP beds available or not | -0.229* | -0.371*** | 0.060 | -0.086* |  |  |  |  |  |  |  |  |
|  | （0.122) | (0.127) | (0.040) | (0.051) |  |  |  |  |  |  |  |  |
| Ratio of VIP beds |  |  |  |  | -3.161** | -4.943*** | 1.260* | -0.286 |  |  |  |  |
|  |  |  |  |  | (1.524) | (1.433) | (0.698) | (0.824) |  |  |  |  |
| Ln Number of VIP beds |  |  |  |  |  |  |  |  | -0.104** | -0.181*** | 0.037* | -0.025 |
|  |  |  |  |  |  |  |  |  | (0.049) | (0.050) | (0.019) | (0.018) |
| Outside environment characteristics | YES | YES | YES | YES | YES | YES | YES | YES | YES | YES | YES | YES |
| Medical institution's external characteristics | YES | YES | YES | YES | YES | YES | YES | YES | YES | YES | YES | YES |
| Medical institution policy-induced | YES | YES | YES | YES | YES | YES | YES | YES | YES | YES | YES | YES |
| Year fixed-effect | YES | YES | YES | YES | YES | YES | YES | YES | YES | YES | YES | YES |
| Observations | 416 | 416 | 416 | 416 | 416 | 416 | 416 | 416 | 416 | 416 | 416 | 416 |

Note: Standard errors are in parentheses.

* p < 0.1, ** p < 0.05, *** p < 0.01

**Table 6-3 Robust check, balanced data**

|  | TFP | Thereof: | | | TFP | Thereof: | | | TFP | Thereof: | | |
| --- | --- | --- | --- | --- | --- | --- | --- | --- | --- | --- | --- | --- |
|  |  | PEFF | TECH | SC |  | PEFF | TECH | SC |  | PEFF | TECH | SC |
|  | (1) | (2) | (3) | (4) | (5) | (6) | (7) | (8) | (9) | (10) | (11) | (12) |
| **Two-way FE model** |  |  |  |  |  |  |  |  |  |  |  |  |
| VIP beds available or not | -0.163** | -0.020 | -0.067* | -0.046 |  |  |  |  |  |  |  |  |
|  | （0.070) | (0.052) | (0.040) | (0.028) |  |  |  |  |  |  |  |  |
| Ratio of VIP beds |  |  |  |  | -3.039** | -1.123 | -1.236*** | -0.301 |  |  |  |  |
|  |  |  |  |  | (1.155) | (0.857) | (0.449) | (0.356) |  |  |  |  |
| Ln Number of VIP beds |  |  |  |  |  |  |  |  | -0.072* | -0.010 | -0.027* | -0.019** |
|  |  |  |  |  |  |  |  |  | (0.037) | (0.023) | (0.015) | (0.009) |
| Outside environment characteristics | YES | YES | YES | YES | YES | YES | YES | YES | YES | YES | YES | YES |
| Medical institution's external characteristics | YES | YES | YES | YES | YES | YES | YES | YES | YES | YES | YES | YES |
| Medical institution policy-induced | YES | YES | YES | YES | YES | YES | YES | YES | YES | YES | YES | YES |
| Year fixed-effect | YES | YES | YES | YES | YES | YES | YES | YES | YES | YES | YES | YES |
| Observations | 375 | 375 | 375 | 375 | 375 | 375 | 375 | 375 | 375 | 375 | 375 | 375 |
| R-Square | 0.293 | 0.258 | 0.228 | 0.246 | 0.293 | 0.259 | 0.228 | 0.243 | 0.294 | 0.258 | 0.227 | 0.246 |
| **Panel Tobit model** |  |  |  |  |  |  |  |  |  |  |  |  |
| VIP beds available or not | -0.072** | -0.009 | -0.040*** | -0.007*** |  |  |  |  |  |  |  |  |
|  | (0.036) | (0.019) | (0.008) | (0.002) |  |  |  |  |  |  |  |  |
| Ratio of VIP beds |  |  |  |  | -2.577*** | -0.853** | -0.913*** | -0.158 |  |  |  |  |
|  |  |  |  |  | (0.401) | (0.389) | (0.185) | (0.181) |  |  |  |  |
| Ln Number of VIP beds |  |  |  |  |  |  |  |  | -0.015*** | 0.010** | -0.011** | -0.003*** |
|  |  |  |  |  |  |  |  |  | （0.009） | （0.005） | （0.005） | （0.000） |
| Outside environment characteristics | YES | YES | YES | YES | YES | YES | YES | YES | YES | YES | YES | YES |
| Medical institution's external characteristics | YES | YES | YES | YES | YES | YES | YES | YES | YES | YES | YES | YES |
| Year fixed effect | YES | YES | YES | YES | YES | YES | YES | YES | YES | YES | YES | YES |
| Individual fixed effect | YES | YES | YES | YES | YES | YES | YES | YES | YES | YES | YES | YES |
| Observations | 375 | 375 | 375 | 375 | 375 | 375 | 375 | 375 | 375 | 375 | 375 | 375 |
| **Simarwilson Estimation results (Bootstrapped 2000 reps)** | | | | | |  |  |  |  |  |  |  |
| VIP beds available or not | -0.265* | -0.253** | -0.042 | -0.211** |  |  |  |  |  |  |  |  |
|  | （0.131) | (0.092) | (0.037) | (0.081) |  |  |  |  |  |  |  |  |
| Ratio of VIP beds |  |  |  |  | -5.411*** | -3.723*** | -0.223 | -0.452 |  |  |  |  |
|  |  |  |  |  | (1.610) | (0.982) | (0.457) | (1.234) |  |  |  |  |
| Ln Number of VIP beds |  |  |  |  |  |  |  |  | -0.137** | -0.135*** | -0.006 | -0.062* |
|  |  |  |  |  |  |  |  |  | (0.049) | (0.036) | (0.015) | (0.029) |
| Outside environment characteristics | YES | YES | YES | YES | YES | YES | YES | YES | YES | YES | YES | YES |
| Medical institution's external characteristics | YES | YES | YES | YES | YES | YES | YES | YES | YES | YES | YES | YES |
| Medical institution policy-induced | YES | YES | YES | YES | YES | YES | YES | YES | YES | YES | YES | YES |
| Year fixed-effect | YES | YES | YES | YES | YES | YES | YES | YES | YES | YES | YES | YES |
| Observations | 375 | 375 | 375 | 375 | 375 | 375 | 375 | 375 | 375 | 375 | 375 | 375 |

Note: Standard errors are in parentheses.

* p < 0.1, ** p < 0.05, *** p < 0.01

TECH: Technical; PEFF: Pure Efficiency; SC: Scale.

**Table 7 Heterogeneity analysis**

|  | | Two-way FE | | | | | | | Panel Tobit | | | | | |
| --- | --- | --- | --- | --- | --- | --- | --- | --- | --- | --- | --- | --- | --- | --- |
|  | | Low/Value=0 | | | | High/Value=1 | | | Low/Value=0 | | | High/Value=1 | | |
|  | (1) | | | (2) | (3) | (4) | (5) | (6) | (7) | (8) | (9) | (10) | (11) | (12) |
| **Economic development level grouping (Panel A)** | | | | | |  |  |  |  |  |  |  |  |  |
| VIP beds available or not | | -0.171 |  | |  | -0.334** |  |  | -0.002 |  |  | -0.253*** |  |  |
|  | | (0.167) |  | |  | (0.146) |  |  | (0.079) |  |  | (0.061) |  |  |
| Ratio of VIP beds | |  | -3.057* | |  |  | -8.777 |  |  | -2.035** |  |  | -3.851*** |  |
|  | |  | (1.667) | |  |  | (5.451) |  |  | (1.036) |  |  | (0.876) |  |
| Ln Number of VIP beds | |  |  | | -0.137 |  |  | -0.106* |  |  | 0.010 |  |  | -0.070*** |
|  | |  |  | | (0.094) |  |  | (0.063) |  |  | (0.025) |  |  | (0.022) |
| Outside environment characteristics | | YES | YES | | YES | YES | YES | YES | YES | YES | YES | YES | YES | YES |
| Medical institution external characteristics | | YES | YES | | YES | YES | YES | YES | YES | YES | YES | YES | YES | YES |
| Medical institution policy induced | | YES | YES | | YES | YES | YES | YES | YES | YES | YES | YES | YES | YES |
| Year fixed effect | | YES | YES | | YES | YES | YES | YES | YES | YES | YES | YES | YES | YES |
| Individual fixed effect | | YES | YES | | YES | YES | YES | YES | YES | YES | YES | YES | YES | YES |
| Observations | | 193 | 193 | | 193 | 210 | 210 | 210 | 193 | 193 | 193 | 210 | 210 | 210 |
| R-Square | | 0.348 | 0.350 | | 0.352 | 0.318 | 0.315 | 0.315 |  |  |  |  |  |  |
| **Medical resource level grouping (Panel B)** | | | | | |  |  |  |  |  |  |  |  |  |
| VIP beds available or not | | -0.098 |  | |  | -0.335* |  |  | -0.086* |  |  | -0.223*** |  |  |
|  | | (0.106) |  | |  | (0.048) |  |  | (0.048) |  |  | (0.025) |  |  |
| Ratio of VIP beds | |  | -4.320* | |  |  | -7.979 |  |  | -3.502*** |  |  | -3.304*** |  |
|  | |  | (2.399) | |  |  | (5.671) |  |  | (0.936) |  |  | (0.473) |  |
| Ln Number of VIP beds | |  |  | | -0.063 |  |  | -0.093 |  |  | -0.034* |  |  | -0.040** |
|  | |  |  | | (0.057) |  |  | (0.068) |  |  | (0.018) |  |  | (0.018) |
| Outside environment characteristics | | YES | YES | | YES | YES | YES | YES | YES | YES | YES | YES | YES | YES |
| Medical institution external characteristics | | YES | YES | | YES | YES | YES | YES | YES | YES | YES | YES | YES | YES |
| Medical institution policy induced | | YES | YES | | YES | YES | YES | YES | YES | YES | YES | YES | YES | YES |
| Year fixed effect | | YES | YES | | YES | YES | YES | YES | YES | YES | YES | YES | YES | YES |
| Individual fixed effect | | YES | YES | | YES | YES | YES | YES | YES | YES | YES | YES | YES | YES |
| Observations | | 215 | 215 | | 215 | 189 | 189 | 189 | 215 | 215 | 215 | 189 | 189 | 189 |
| R-Square | | 0.319 | 0.325 | | 0.321 | 0.340 | 0.337 | 0.337 |  |  |  |  |  |  |
| **Ownership (Panel C)** | | | | | |  |  |  |  |  |  |  |  |  |
| VIP beds available or not | | 0.000 |  | |  | -0.128* |  |  | 0.041 |  |  | -0.139** |  |  |
|  | | (.) |  | |  | (0.069) |  |  | (0.037) |  |  | (0.065) |  |  |
| Ratio of VIP beds | |  | -71.297** | |  |  | -1.289* |  |  | -4.475*** |  |  | -2.312** |  |
|  | |  | (28.733) | |  |  | (0.756) |  |  | (0.924) |  |  | (0.936) |  |
| Ln Number of VIP beds | |  |  | | -1.188** |  |  | -0.040 |  |  | -0.025 |  |  | -0.014 |
|  | |  |  | | (0.476) |  |  | (0.028) |  |  | (0.021) |  |  | (0.014) |
| Outside environment characteristics | | YES | YES | | YES | YES | YES | YES | YES | YES | YES | YES | YES | YES |
| Medical institution external characteristics | | YES | YES | | YES | YES | YES | YES | YES | YES | YES | YES | YES | YES |
| Medical institution policy induced | | YES | YES | | YES | YES | YES | YES | YES | YES | YES | YES | YES | YES |
| Year fixed effect | | YES | YES | | YES | YES | YES | YES | YES | YES | YES | YES | YES | YES |
| Individual fixed effect | | YES | YES | | YES | YES | YES | YES | YES | YES | YES | YES | YES | YES |
| Observations | | 208 | 208 | | 208 | 208 | 208 | 208 | 208 | 208 | 208 | 208 | 208 | 208 |
| R-Square | | 0.378 | 0.371 | | 0.347 | 0.337 | 0.341 | 0.378 |  |  |  |  |  |  |
| **Specialization (Panel D)** | | | | | |  |  |  |  |  |  |  |  |  |
| VIP beds available or not | | -0.112 |  | |  | -0.501*** |  |  | -0.071*** |  |  | -0.472*** |  |  |
|  | | (0.141) |  | |  | (0.173) |  |  | (0.021) |  |  | (0.010) |  |  |
| Ratio of VIP beds | |  | -3.789 | |  |  | -5.851** |  |  | -2.967*** |  |  | -6.543*** |  |
|  | |  | (4.291) | |  |  | (2.319) |  |  | (0.521) |  |  | (1.277) |  |
| Ln Number of VIP beds | |  |  | | -0.054 |  |  | -0.268** |  |  | -0.025*** |  |  | -0.285*** |
|  | |  |  | | (0.054) |  |  | (0.104) |  |  | (0.008) |  |  | (0.002) |
| Outside environment characteristics | | YES | YES | | YES | YES | YES | YES | YES | YES | YES | YES | YES | YES |
| Medical institution external characteristics | | YES | YES | | YES | YES | YES | YES | YES | YES | YES | YES | YES | YES |
| Medical institution policy induced | | YES | YES | | YES | YES | YES | YES | YES | YES | YES | YES | YES | YES |
| Year fixed effect | | YES | YES | | YES | YES | YES | YES | YES | YES | YES | YES | YES | YES |
| Individual fixed effect | | YES | YES | | YES | YES | YES | YES | YES | YES | YES | YES | YES | YES |
| Observations | | 264 | 264 | | 264 | 152 | 152 | 152 | 264 | 264 | 264 | 152 | 152 | 152 |
| R-Square | | 0.305 | 0.305 | | 0.433 | 0.422 | 0.429 | 0.305 |  |  |  |  |  |  |

Cluster standard errors in parentheses* p<0.1, ** p<0.05, *** p<0.01
